# Molecular Detection of Zoonotic and Veterinary Pathogenic Bacteria in Pet Dogs and Their Parasitizing Ticks in Junggar Basin, North-Western China

**DOI:** 10.3389/fvets.2022.895140

**Published:** 2022-07-08

**Authors:** Jia Guo, Shengnan Song, Shuzhu Cao, Zhihua Sun, Qiyue Zhou, Xingmei Deng, Tianyi Zhao, Yingjin Chai, Dexin Zhu, Chuangfu Chen, P. I. Baryshnikov, Hugh T. Blair, Zhen Wang, Yuanzhi Wang, Hui Zhang

**Affiliations:** ^1^State International Joint Research Center for Animal Health Breeding, College of Animal Science and Technology, Shihezi University, Shihezi, China; ^2^College of Pharmacy, Shihezi University, Shihezi, China; ^3^College of Veterinary, Altai National Agricultural University, Barnaul, Russia; ^4^International Sheep Research Center, School of Agriculture and Environment, Massey University, Palmerston North, New Zealand; ^5^School of Medicine, Shihezi University, Shihezi, China

**Keywords:** pet dogs, tick-borne bacteria, *Rhipicephalus turanicus* sensu stricto, north-western China, *Brucella*

## Abstract

Despite the recognized epidemiological importance of ticks as vectors for pathogens that cause numerous zoonotic and veterinary diseases, data regarding the pathogens of pet dogs and their parasitic ticks in the Junggar Basin are scarce. In this study, a total of 178 blood samples and 436 parasitic ticks were collected from pet dogs in Junggar Basin, Xinjiang Uygur Autonomous Region (XUAR), north-western China. All ticks were identified as *Rhipicephalus turanicus* sensu stricto (s.s.) according to morphological and molecular characteristics. *Rh. turanicus* s.s. ticks were collected from pet dogs in China for the first time. Seven tick-borne pathogens, such as *Ehrlichia chaffeensis, Anaplasma phagocytophilum, Rickettsia massiliae, Candidatus* R. barbariae, *Brucella* spp., *Rickettsia sibirica*, and *Anaplasma ovis*, were detected from ticks, whereas the first five bacteria were detected from blood samples of dogs. *Brucella* spp. was the most predominant pathogen in both blood samples and ticks of pet dogs, with the detection rates of 16.29 and 16.74%, respectively. Moreover, 17 ticks and 1 blood sample were co-infected with two pathogens, and 1 tick was co-infected with three pathogens. This study provided molecular evidence for the occurrence of *Anaplasma* spp., *Ehrlichia* spp., *Rickettsia* spp., and *Brucella* spp. circulating in pet dogs and their parasitic ticks in Junggar Basin, north-western China. These findings extend our knowledge of the tick-borne pathogens in pet dogs and their parasitic ticks in Central Asia; therefore, further research on these pathogens and their role in human and animal diseases is required.

## Introduction

Tick-borne diseases, such as zoonotic and veterinary diseases, represent a serious threat to human and/or animal health (1, 2). Globally, *Canis familiaris* (domestic dog) is raised as a pet and shares the household environment with humans; they serve as a host for infected ticks that can be carried into the household environment (3). These ticks may represent a threat to health, especially to children, elder individuals, and immunocompromised individuals (4). Since dogs can be considered as “sentinels” for monitoring the risk of disease affecting humans in an endemic area, the investigation of neglected zoonotic pathogens in pet dogs and the vectors that transmit pathogens is important in the prevention and control of zoonotic diseases (5–7).

Many members of the genus *Brucella* and certain members of the order Rickettsiales (*Rickettsia, Anaplasma*, and *Ehrlichia*) are important zoonotic and veterinary pathogens, causing brucellosis, rickettsiosis, anaplasmosis, and ehrlichiosis, which are considered as re-emerging tick-borne diseases worldwide (2, 8, 9). In the past 30 years, at least 13 emerging tick-borne pathogens that infect humans have been identified in the order Rickettsiales and found to be present in mainland China. Among these species, the most important species *Rickettsia sibirica, Rickettsia conorii, Rickettsia massiliae, Candidatus* R. tarasevichiae, *Rickettsia raoultii, Ehrlichia chaffeensis*, and *Anaplasma phagocytophilum* have been confirmed as the causative agents of human rickettsiosis, human monocytic ehrlichiosis, and human granulocytic anaplasmosis (2, 10–14). Other species prevalent in ticks and dogs included *Anaplasma bovis, Anaplasma platys, Anaplasma ovis, Ehrlichia canis, Ehrlichia ewingii*, and *Rickettsia felis* (15–17). *Brucella* spp. can be classically transmitted to humans *via* inhalation of aerosolized bacteria or *via* ingestion of, or contact with, contaminated tissues or derived products (18, 19). Considering livestock, brucellosis infection relates to direct contact with infected animals through the exchange of body fluids and *via* mating (8). The *Brucella* genus contains 12 valid species, among which *Brucellas melitensis, Brucella abortus, B. canis, Brucella suis*, and *B. ovis* have emerged in China and can infect livestock, wildlife, and humans and are transmitted by ticks and their offspring (8, 20, 21).

Xinjiang Uygur Autonomous Region (XUAR) is the largest province in China, which hosts a wide range of natural-focal diseases, among which brucellosis is the most common tick-borne disease of livestock (22); additionally, emerging tick-borne zoonoses caused by *R. raoultii, E. chaffeensis*, and *A. phagocytophilum* have been reported from sheep (23, 24). The Junggar Basin is located between the Altay Mountain and Tian Shan Mountain in XUAR and is the second-largest inland basin in China. Currently, tick-borne infections in pets in the Junggar Basin have not been studied. Therefore, the aim of the present study was to determine the prevalence of several tick-borne pathogenic bacteria, particularly *Rickettsia, Ehrlichia, Anaplasma*, and *Brucella*, in pet dogs and their ticks in Junggar Basin, XUAR, north-western China.

## Materials and Methods

### Sample Collection

During the period between 2017 and 2020, late April to mid-May (coinciding with the peak activities of adult ticks), blood and tick samples were collected from pet dogs based on clinical symptoms that include but not limited to depression, fever, lethargy, weakness, weight loss, and anorexia at six veterinary clinics close to pastures in Shihezi City (483 m above sea level; 44°27′N 86°06′E) and Shawan City (797 m above sea level; 44°29′N 85°56′E), Junggar Basin, XUAR, north-western China. A total of 178 blood and 436 tick samples were collected from pet dogs. All samples were collected under the permission of the pet owners, and sample collection was performed by local veterinarians. All tick samples correspond to blood samples according to each individual dog. The blood samples were collected into vacutainer tubes that contained ethylenediaminetetraacetic acid (EDTA) anticoagulant, and the ticks were placed in tubes that contained 75% ethanol and 5% glycerine to keep specimens better preserved and stored at −80°C for further possible virus studies.

### DNA Extraction and Identification of Ticks

Total DNA was extracted from 200 μl of whole blood samples using the Blood DNA Extraction Kit (Omega Bio-tek, Norcross, USA) according to the manufacturer's instructions, and genomic DNA from each tick was extracted using the TIANamp Genomic DNA Kit (TIANGEN, Beijing, China). Before DNA extraction, all ticks were identified based on morphology as described previously (25). Subsequently, 45 representative ticks, with 5–8 ticks at each veterinary clinic, were subjected to molecular classification analysis based on partial mitochondrial [*12S rRNA* and cytochrome *c* oxidase subunit 1 (*COI*)] gene sequences to confirm tick species (23).

### Detection of Tick-Borne Pathogens and Sequence Analysis

We used a partial *16S rRNA* gene to detect *Anaplasma* spp., *A. phagocytophilum, Ehrlichia* spp., and *E. chaffeensis*, as described previously (26–29). The molecular detection of *Rickettsia* was performed using the citrate synthase (*gltA*) and outer membrane protein B (*ompB*) genes (30). *A. ovis* and *E. canis* were detected based on the major surface protein 4 (*msp4*) gene (26) and *gltA* gene (31), respectively. *Brucella* spp. were identified using the partial *omp22* gene encoding 22-kD outer membrane protein (8). The DNA of *Anaplasma, Ehrlichia, Rickettsia*, and *Brucella* amplified in our laboratory was used as positive controls. Double-distilled water was used as a negative control (Dongsheng, Guangzhou, China). The amplified products were cloned into the pGEM-T Easy vector (TransGen Biotech, Beijing, China), according to the instructions, and then sequenced.

The sequence results were compared with the reference sequences available in centralized databases using a basic local alignment search tool (BLAST) (http://www.ncbi.nlm.nih.gov/BLAST/). Phylogenetic trees were constructed using the maximum-likelihood method using MEGA X software (https://www.megasoftware.net).

### Statistical Analysis

Statistical analyses were performed using GraphPad Prism 7 software (GraphPad, Inc., La Jolla, CA, USA), one-tailed or *t*-test was used to determine the differences, and the data were expressed as mean values ± standard deviation (SD). The association of pathogen DNA between dogs and ticks was computed using the MedCalc Statistical Software. *p-values* <0.05 were considered statistically significant.

## Results

**Figure 1:**
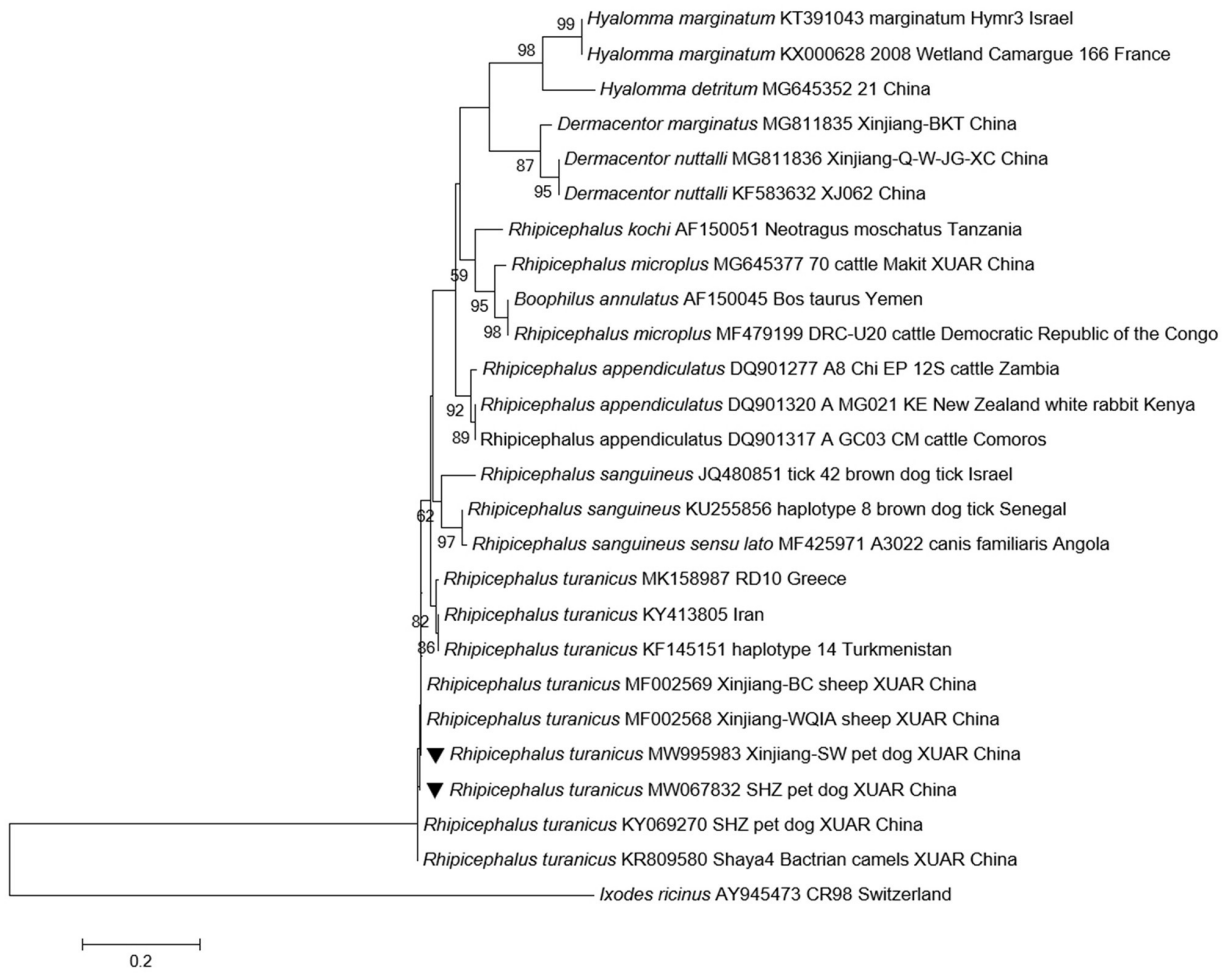
Phylogenetic tree based on *12S rRNA* sequences of ticks collected from pet dogs. New sequences obtained in this study are indicated by black triangles.

All ticks (186 male ticks and 250 female ticks) were identified as *Rhipicephalus turanicus* sensu stricto (s.s.) based on their morphology. The sequencing data based on BLAST analyses for *12S rRNA* and *COI* of ticks confirmed the morphological identification. Morphological analyses are shown in Supplementary Figure S1, and phylogenetic analyses are shown in Figure 1 and Supplementary Figure S2. The obtained sequences of *Rh*. *turanicus* s.s. have been deposited in the GenBank database (*12S rRNA*: MW067832 and MW995983; *COI*: MW065551 and MZ026893).

Seven tick-borne pathogenic bacteria were detected in the ticks, out of which *Brucella* spp. were the most prevalent pathogens with a detection rate of 16.74%, followed by *E. chaffeensis* (8.26%), *R. massiliae* (6.42%), *A. phagocytophilum* (5.05%), *Candidatus* R. barbariae (4.13%), *R. sibirica* (3.21%), and *A. ovis* (0.92%; Table 1). Among them, 18 ticks were co-infected (17 ticks were co-infected with two pathogens and 1 tick was co-infected with three pathogens; Table 2). Meanwhile, we found that 5 tick-borne pathogens were detected in 178 blood samples; among them, *Brucella* spp. was most prevalent with a detection rate of 16.29%, followed by *E. chaffeensis* (5.62%), *A. phagocytophilum* (4.49%), *Candidatus* R. barbariae (1.69%), and *R. massiliae* (1.12%; Table 1). Moreover, *E. chaffeensis* and *Brucella* spp. were simultaneously detected in 1 blood sample. The overall detection rate of tick-borne pathogenic bacteria in ticks was significantly higher than that in blood samples of pet dogs (*p* < 0.0001). All ticks and blood samples were screened for the presence of *E. canis*; however, none of the samples was infected with this bacterium.

**Table 1:** Detection of *Anaplasma, Ehrlichia, Rickettsia*, and *Brucella* in blood and tick samples collected from pet dogs in Shihezi and Shawan, Xinjiang Uygur Autonomous Region, north-western China.

**Sample type**	**Sample site**	**No**.	***Anaplasma* species, No. (%) positive**	***Ehrlichia* species, No. (%) positive**	***Rickettsia* species, No. (%) positive**	***Brucella* spp., No. (%) positive**
Tick	Shawan	329	*A. phagocytophilum*, 17 (5.17)	*E. chaffeensis*, 25 (7.60)	*R. massiliae*, 20 (6.08)	*Brucella* spp., 58 (17.63)
					*R. sibirica*, 9 (2.74)	
					*Candidatus* R. barbariae, 6 (1.82)	
	Shihezi	107	*A. phagocytophilum*, 5 (4.67)	*E. chaffeensis*, 11 (10.28)	*Candidatus* R. barbariae, 12 (11.21)	*Brucella* spp., 15 (14.02)
			*A. ovis*, 4 (3.74)		*R. sibirica*, 5 (4.67)	
					*R. massiliae*, 8 (7.48)	
	Total	436	*A. phagocytophilum*, 22 (5.05)	*E. chaffeensis*, 36 (8.26)	*Candidatus* R. barbariae, 18 (4.13)	*Brucella* spp., 73 (16.74)
			*A. ovis*, 4 (0.92)		*R. massiliae*, 28 (6.42)	
					*R. sibirica*, 14 (3.21)	
Blood	Shawan	117	*A. phagocytophilum*, 5 (4.27)	*E. chaffeensis*, 7 (5.98)	*Candidatus* R. barbariae, 3 (2.56)	*Brucella* spp., 21 (17.95)
					*R. massiliae*, 2 (1.71)	
	Shihezi	61	*A. phagocytophilum*, 3 (4.92)	*E. chaffeensis*, 3 (4.92)		*Brucella* spp., 8 (13.11)
	Total	178	*A. phagocytophilum*, 8 (4.49)	*E. chaffeensis*, 10 (5.62)	*Candidatus* R. barbariae, 3 (1.69)	*Brucella* spp., 29 (16.29)
					*R. massiliae*, 2 (1.12)	

**Table 2:** Co-infection of pathogens in ticks and blood of pet dogs in this study.

**Sample type (n)**	**No. (%) of samples infected with**					
	**Two pathogens**					**Three pathogens**
	**Bsp+Ech**	**Ech+Aph**	**Ech+Rma**	**Ech+Can**		**Bsp+Ech+Aov**
Tick (436)	3 (0.69)	6 (1.38)	4 (0.92)	4 (0.92)		1 (0.23)
Blood (178)	1 (0.56)	0	0	0		0

*n, number; Bsp, Brucella spp., Ech, Ehrlichia chaffeensis; Aph, Anaplasma phagocytophilum; Rma, Rickettsia massiliae; Can, “Candidatus Rickettsia barbariae”; Aov, Anaplasma ovis*.

Among all the positive ticks and blood samples, *Anaplasma* spp. and *Brucella* spp. showed 99.23–100% and 99.6–100% identity to the corresponding sequence of *Anaplasma* sp. BL102-7 (KJ410249) from XUAR, China and *Brucella* sp. YC31 (MK201679) from XUAR, China, respectively. *Ehrlichia* spp. showed 99.64–99.65% identity to the corresponding sequence of *Ehrlichia* sp. QYP9 (KY630175) from Anhui Province, China and *Ehrlichia sp*. XJ-Eh1 (MF098393) from XUAR, China. *A. phagocytophilum* and *E. chaffeensis* showed 99.84% and 99.23–100% identity to the corresponding sequence of *A. phagocytophilum* (KJ782386) from XUAR, China and *E. chaffeensis* (MN368552) from Egypt respectively. The msp4 sequences (MW802667) showed 100% identity to the msp4 sequence of *A. ovis* (MN198191) from China. In addition, based on *gltA* gene and *ompB* gene, the sequences of *R. massiliae, Candidatus* R. barbariae, and *R. sibirica* achieved 99.83–100, 99.76–100, and 99.38–100% similarities with the corresponding sequences of available in GenBank, respectively. The GenBank accession numbers are shown in Supplementary Table S1.

## Discussion

Tick-borne infections, especially zoonotic diseases, have been increasing in humans and pet dog cases (15). However, a few studies have analyzed pet dogs and their ticks in the Junggar Basin, north-western China. Herein, we detected *Brucella* DNA in pet dog-associated ticks in Junggar Basin. Moreover, we identified three *Rickettsia* species (*R. massiliae, R. sibirica*, and *Candidatus* R. barbariae), two *Anaplasma* species (*A. phagocytophilum* and *A. ovis*), and the *Ehrlichia* species *E. chaffeensis* from pet dogs and their *Rh. turanicus* s.s. ticks. The spread of these pathogens in the human household environment increases the range of vectors and reservoirs of tick-borne pathogens and provides a basis for assessing the risk of infection in humans.

The livestock industry is one of the main sources of its economic growth in XUAR (32). Almost every sheep farm likes to keep one dog for guarding their belongings (17). Most dogs live around the pasture and their owners, so it is a common phenomenon for these dogs to be bitten by ticks from livestock or to be parasitized by free-living ticks from pastures. *Rh. turanicus* is widely distributed in Central Asia, North Africa, and Europe and is the dominant tick species in XUAR (23, 33). Previous studies have reported that *Rh. turanicus* is parasitic in dogs and represents a risk for transmission of pathogens, such as *Rickettsia, Anaplasma*, and *Ehrlichia* in Israel, Italy, Greece, and Turkmenistan (34, 35), and serves as a bridge vector for humans (30). However, in all previous studies that infested pet dogs in XUAR, only *Rh. sanguineus* sensu lato has been reported, while *Rh. turanicus* s.s., the dominant tick species, has not been reported, it may be due to the following reasons: i) tick-borne diseases in pet dogs have not previously received much attention in XUAR and ii) it is possible that earlier misidentification that usually happens in *Rh. sanguineus* s.l. complex, so it might be found earlier but due to misidentification, it was reported as *Rh. sanguineus*; however, in fact, it was *Rh. turanicus* s.s. tick.

**Figure 2:**
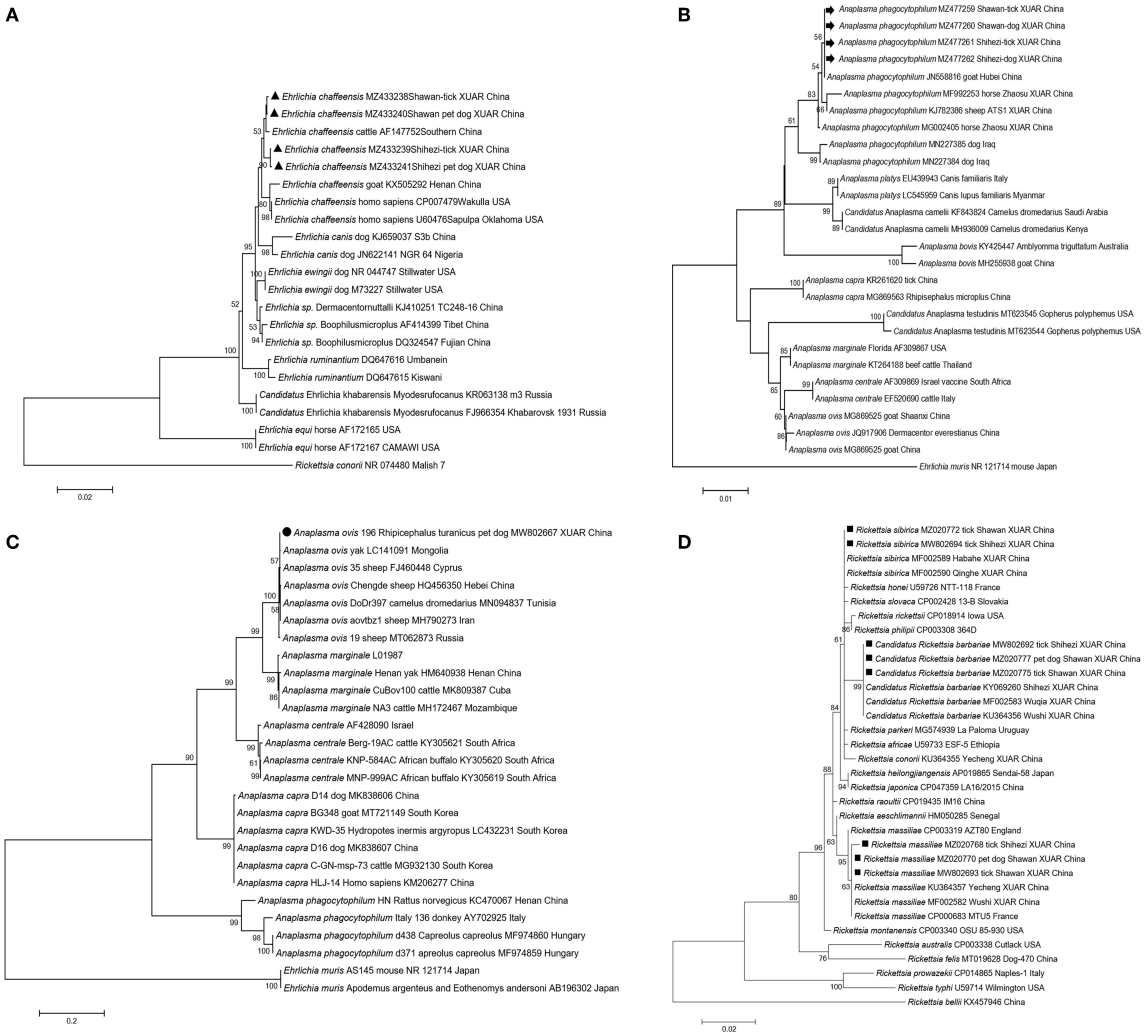
Phylogenetic trees of tick-borne pathogenic bacteria detected from pet dogs and their associated ticks from Shihezi and Shawan, Junggar Basin, Xinjiang Uygur Autonomous Region, north-western China. The evolutionary history was inferred *via* the maximum-likelihood method (bootstrap replicates: 1,000) using MEGA X. New sequences obtained in this study are indicated in black triangles for *Ehrlichia chaffeensis*, black arrows for *Anaplasma phagocytophilum*, black circle for *Anaplasma ovis*, black squares for three *Rickettsia* species (*Rickettsia massiliae, Rickettsia sibirica*, and *Candidatus* R. barbariae) based on *gltA* gene respectively. **(A)** *Ehrlichia chaffeensis* based on *16S rRNA* gene; **(B)** *Anaplasma phagocytophilum* based on *16S rRNA* gene; **(C)** *Anaplasma ovis* based on *msp4* gene; **(D)** *Rickettsia massiliae, Rickettsia sibirica*, and *Candidatus* R. barbariae based on *gltA* gene.

*Anaplasma phagocytophilum* and *E. chaffeensis* are considered as emerging pathogens of public health importance as they can infect humans; they are naturally maintained in tick-mammal cycles and have been detected in ticks, rodents, deer, domestic animals, and humans (36–40). *A. phagocytophilum* and *E. chaffeensis* have been previously detected in domestic animals and their *Rh. turanicus* s.s. ticks in XUAR (41). In this study, we detected these two pathogens in neglected pet dogs and their parasitic ticks. Phylogenetic relationships showed that *A. phagocytophilum* and *E. chaffeensis* detected from pet dogs and their ticks formed a cluster with strains detected from livestock in southern China or other counties/cities in XUAR (Figures 2A,B). *Rh. turanicus* s.s. is a three-host tick and is characterized by constant host changes during development (42); this suggests that there is a possibility of pathogen transmission from domestic animals to pet dogs *via* ticks.

Canine monocytic ehrlichiosis (CME), a pathogen caused by *E. canis*, has been found in dogs and ticks in North America, Europe, Asia, and Africa since it was first identified in Algeria in 1935 and is now spreading around the world (15, 43–45). One recent study in XUAR reported that the prevalence of *E. canis* in pet dogs was 12.12% (45), while another study did not detect *Ehrlichia* spp. in *Rh. sanguineus* s.l. from XUAR when detecting dogs for vector-borne agents in 10 provinces (46), which is consistent with the detected in this study. These differences in prevalence may be attributed to variations in techniques used, sources, and numbers of samples (34).

*Anaplasma ovis* has been identified in China and worldwide for many years since its first description (47, 48). *A. ovis* has been previously detected from ticks or blood samples from livestock and wild animals over ten counties in XUAR (22, 47, 49). In this study, *A. ovis* was only detected in *Rh. turanicus* s.s. obtained from pet dogs (Figure 2C), all blood samples of pet dogs were tested negative. However, based on a report that a strain isolated from a stray dog in Henan has been shown to be highly homologous to the *A. ovis* detected in a human in Cyprus (17), it suggests the possibility that ticks, dogs, and even humans can be infected with *A. ovis*. Therefore, individuals who have been in contact with animals infected with the pathogen or those caring for dogs may be at a risk.

At present, at least 19 validated spotted fever group (SFG) *Rickettsia* species have been detected in ticks in China (50). *R. sibirica, R. massiliae*, and *Candidatus* R. barbariae, which had previously been detected in *Rh. turanicus* s.s. ticks obtained from sheep in XUAR (23, 51), were found in the same tick species and/or blood samples obtained from pet dogs in this study (Figure 2D and Supplementary Figure S3). The detection rate of *Rickettsia* in the ticks obtained from pet dogs was lower than that in the ticks obtained from domestic in XUAR (22, 23). Additionally, the overall detection rate of *Rickettsia* infection in ticks (13.76%) was significantly higher than that in blood samples of pet dogs (2.81%). As SFG *Rickettsia* is endemic in north-western China, human rickettsiosis cases have been reported in the recent years in this area, the fact that the Rickettsia species were detected from pet dogs in this study suggests that surveillance of pathogens and ticks in pet dogs is needed to clarify the risk level and prevent human infection.

Brucellosis, also known as Malta fever in humans, can lead to abortion in livestock (52). Its pathogen *Brucella* spp. may be carried continuously through transstadial transmission of ticks (engorged adult female ticks, eggs, and larvae) and transmitted to healthy animals *via* blood sucking (8, 53). In spite of evidence showing that *Brucella* spp. can be transmitted by ticks (53), but no follow-up survey has confirmed such transmission. In 2018, Wang et al. (8) found that *B. melitensis* and *B. abortus* can be transmitted vertically in *Dermacentor marginatus* obtained from sheep. After that, *Brucella* DNA was successively identified in *D. marginatus, Dermacentor nuttalli, Hyalomma asiaticum, Haemaphysalis punctata, Haemaphysalis longicornis*, and *Rh. turanicus* s.s. obtained from livestock and/or free-living ticks in XUAR and Henan Province, China (22, 42, 54). In the present study, *Brucella* DNA was detected from blood samples and *Rh. turanicus* s.s. ticks infesting pet dogs. This may suggest the role of *Rh. turanicus* s.s. ticks in the transmission of *Brucella* spp. in the Junggar Basin. Moreover, since pet dogs are considered as human companions and share the household environment with humans when ticks parasitize pet dogs, it is suggested that both dogs and their owners are at risk of contracting brucellosis *via* tick bites. This finding indicates that in addition to the prevention of *Brucella* transmission through classical routes, we also need to strengthen the prevention of the transmission of *Brucella* by ticks from pet dogs. Moreover, the most important species to infect dogs are *B. melitensis, B. abortus, B. canis*, and *B. suis* in China (20, 55, 56), so further studies will be necessary to confirm the *Brucella* species by isolation and identification, to improve the understanding of the epidemiology of these tick-borne diseases, and to monitor emerging tick-borne pathogens and factors influencing their prevalence, which will facilitate implementing integrated strategies for controlling ticks and tick-borne pathogens in China.

In addition, we found two pathogens (*Brucella* and *Ehrlichia*) that might share common tick vectors and reservoir pet dogs. Interestingly, the two pathogen sequences retrieved from positive ticks were identical to those found in their dog host, which may mean that ticks are vectors for the two pathogens or the presence of the two pathogens in ticks was due to the presence of the pathogens in the blood meal (57). We also detected co-infection of two or three bacteria in pet dogs and/or their parasitic ticks in this study. These bacteria share a common tick vector, and pet dogs may become infected with these pathogens either simultaneously or sequentially (58). Although the influence of co-infection on disease severity remains unclear (59), it may result in more complicated pathogenicity and worse prognosis if humans or pet dogs are parasitized by these ticks (60). Therefore, additional efforts should be made to actively monitor the prevalence of pathogenic and potentially pathogenic tick-borne bacteria in pet dogs and their owners in XUAR and China to assess the risk of infection in pets and humans.

## Data Availability Statement

The datasets presented in this study can be found in online repositories. The names of the repository/repositories and accession number(s) can be found in the article/Supplementary Material.

## Ethics Statement

The animal study was reviewed and approved by the Animal Health Committee of Shihezi University. Written informed consent was obtained from the owners for the participation of their animals in this study.

## Author Contributions

JG, HZ, YW, and ZW conceived and designed the study. JG, SS, and SC critically revised the manuscript. HZ and JG analyzed the data and drafted the manuscript. ZS and QZ conducted the morphological test of dog ticks. CC, XD, TZ, YC, DZ, PB, and HB conducted molecular analyses. All authors contributed to the article and approved the submitted version.

## Funding

This research was supported by the Scientific and Technological Tackling Plan for Key Fields of the Corps (grant nos. 2022DB018 and 2021AB012), the National Natural Science Foundation of China (grant nos. 31860691 and 31602080), the International Science and Technology Cooperation Promotion Plan (grant nos. 2015DFR31110 and GJHZ201709), the Training Program for Excellent Young Teachers Colleges and Universities of Corps (grant no. CZ027202), and the Youth Science and Technology Innovation Leading Talent Program of Corps (grant no. 2017CB002).

## Conflict of Interest

The authors declare that the research was conducted in the absence of any commercial or financial relationships that could be construed as a potential conflict of interest.

## Publisher's Note

All claims expressed in this article are solely those of the authors and do not necessarily represent those of their affiliated organizations, or those of the publisher, the editors and the reviewers. Any product that may be evaluated in this article, or claim that may be made by its manufacturer, is not guaranteed or endorsed by the publisher.

## Supplementary Material

Supplementary Figure 1 The morphological characteristics of male ticks collected from pet dogs.

Supplementary Figure 2 Phylogenetic tree based on cytochrome *c* oxidase subunit 1(*COI*) sequences of ticks collected from pet dogs. New sequences obtained in this study are indicated by white triangles.

Supplementary Figure 3 Phylogenetic tree of *Rickettsia* species based on *ompB* gene within pet dogs and their ticks. New sequences obtained in this study are indicated in black diamonds.

Supplementary Table 1 GenBank accession numbers of representative nucleotide sequences.
