# Supplementary material for: Molecular Detection of Zoonotic and Veterinary Pathogenic Bacteria in Pet Dogs and Their Parasitizing Ticks in Junggar Basin, North-Western China
Source: Front Vet Sci. 2022 Jul 8;9:895140. doi: 10.3389/fvets.2022.895140 (PMC9311330; doi:10.3389/fvets.2022.895140)
Supplement: Supplementary Figure 1 — The morphological characteristics of male ticks collected from pet dogs. [file Table_1.DOCX]

**Supplementary Figure 1** **|** Morphological characteristic of male ticks collected from pet dogs.

**
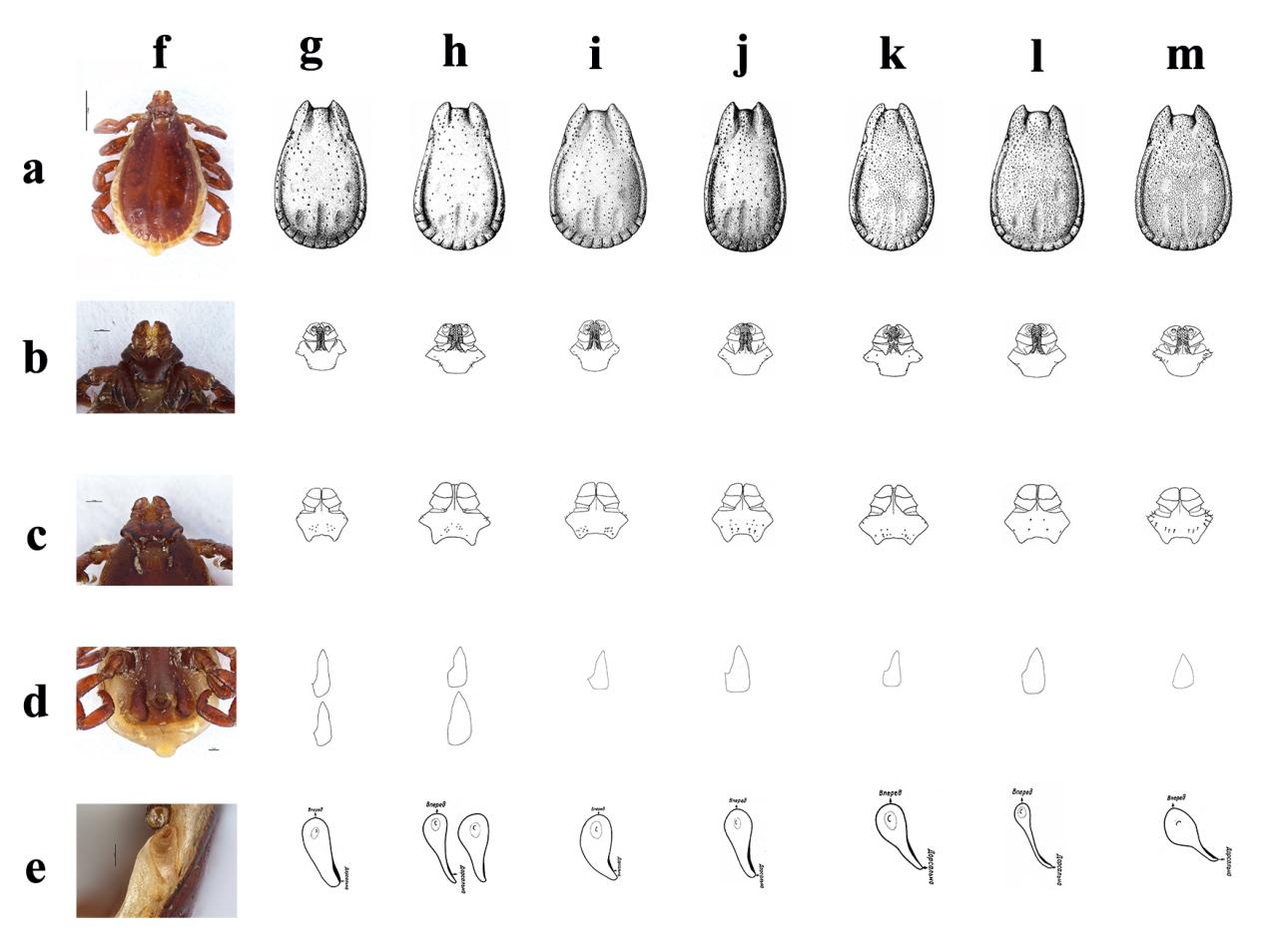
**

The dorsal posterolateral furrow of the *Rh. turanicus* sensu stricto (s.s.) is ovoid, while the other ticks are longer or smaller than that. The shape of the spiracular plate is approximately oblong, which is obviously different from the other ticks which have a more pointed end to the spiracular plate. The shape of the adanal plate from *Rh. turanicus* s.s. is longer and narrower, which is different from the triangular or wider features of other ticks. **a:** Dorsal view; **b:** Basis capituli, dorsal view; **c:** Basis capituli, ventral view; **d:** adanal plate; **e:** spiracular plate; **f:** *Rh. turanicus* s.s. in this study; **g:** *Rh. turanicus*, **h:** *Rh. sanguineus*, **i:** *Rh. rossicus*, **j:** *Rh. pumilio*, **k:** *Rh. schulzei*, **l:** *Rh. leporis*, **m:** *Rh. bursa.*

Note: g-m were obtained from Filippova's book (Filippova N.A., 1997).
