# Supplementary figures and images for: Molecular Detection of Zoonotic and Veterinary Pathogenic Bacteria in Pet Dogs and Their Parasitizing Ticks in Junggar Basin, North-Western China
Source: Front Vet Sci. 2022 Jul 8;9:895140. doi: 10.3389/fvets.2022.895140 (PMC9311330; doi:10.3389/fvets.2022.895140)

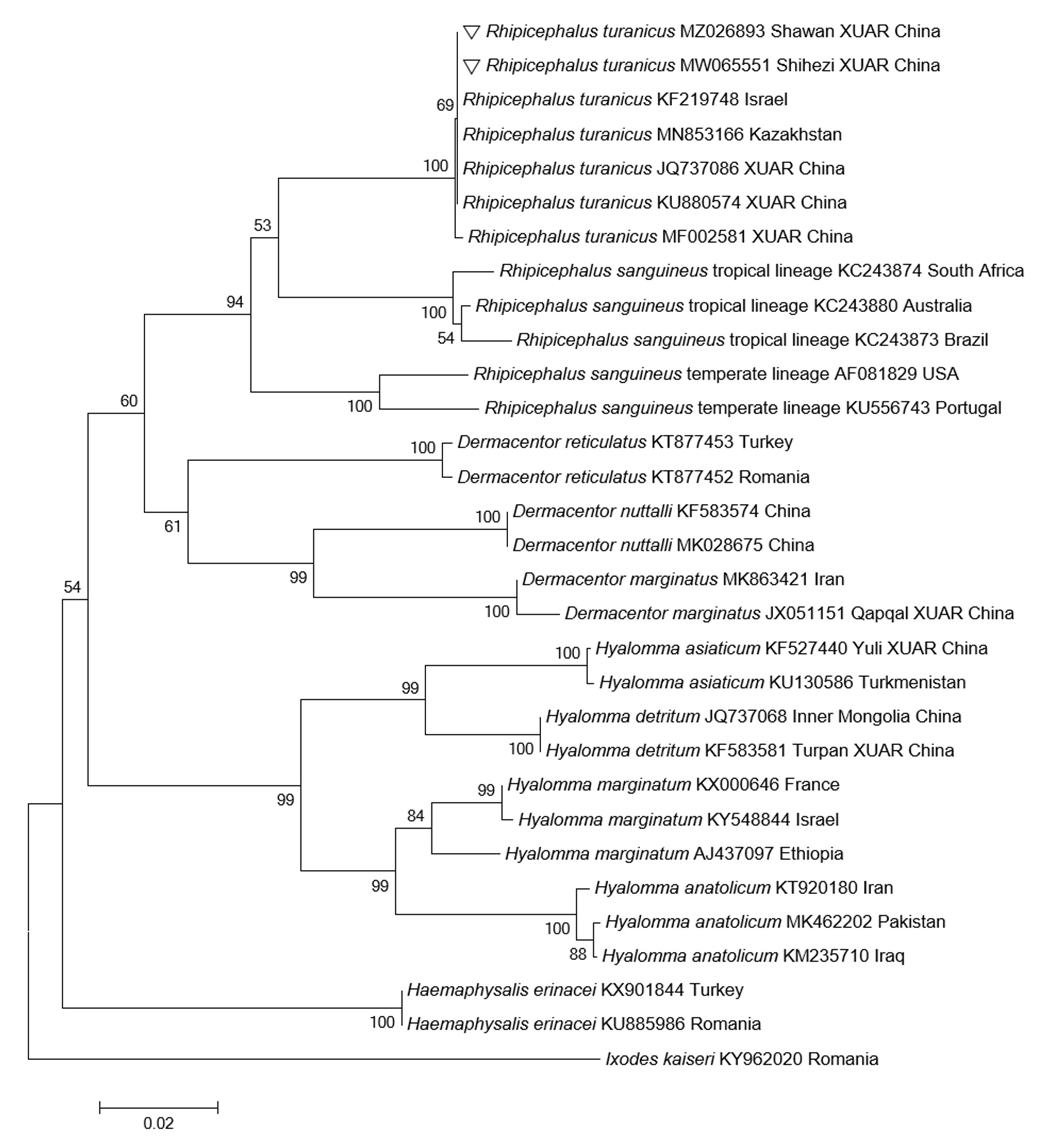

Supplement: Supplementary Figure 2 — Phylogenetic tree based on cytochrome c oxidase subunit 1(COI) sequences of ticks collected from pet dogs. New sequences obtained in this study are indicated by white triangles. [file Image_1.JPEG]

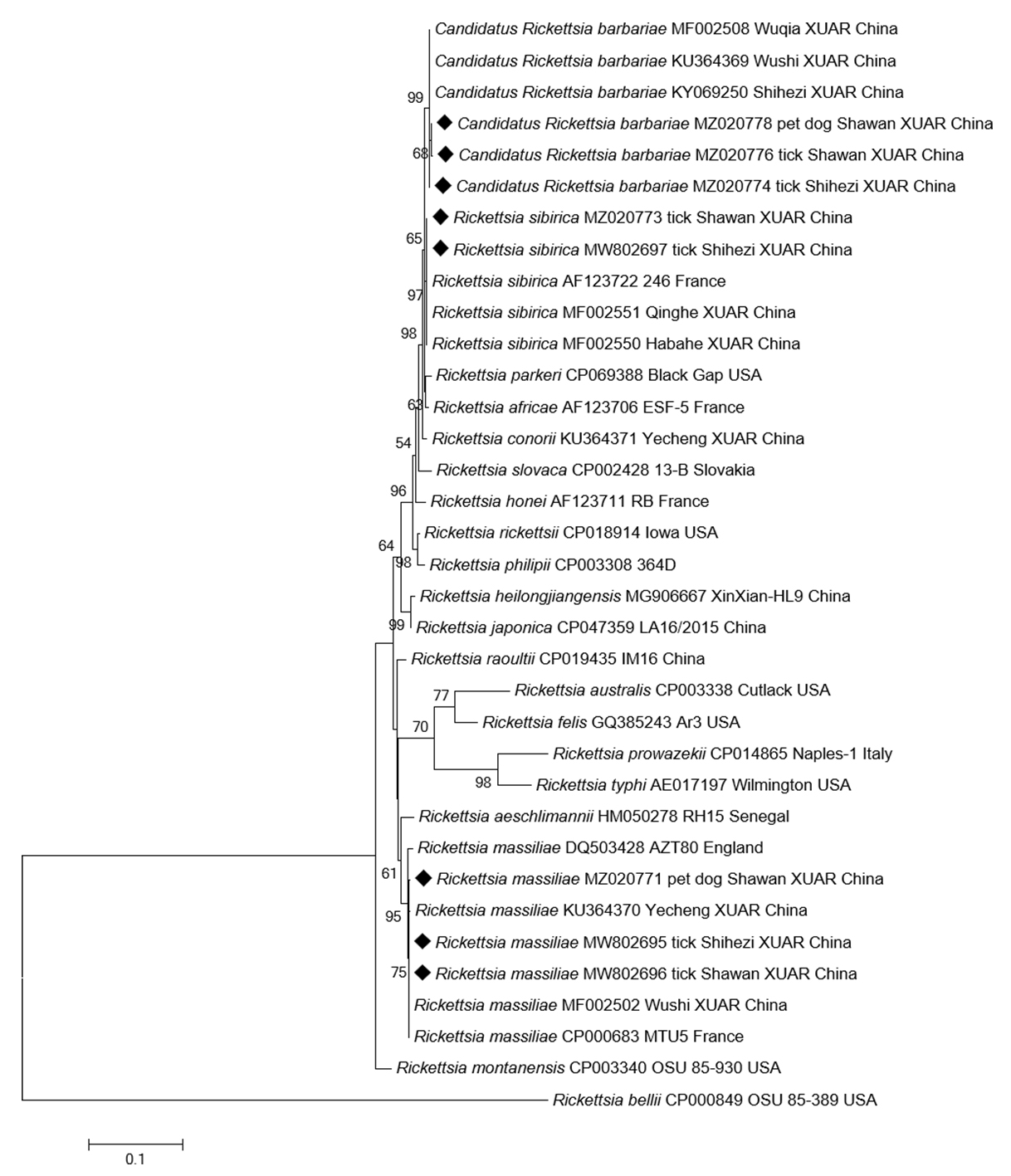

Supplement: Supplementary Figure 3 — Phylogenetic tree of Rickettsia species based on ompB gene within pet dogs and their ticks. New sequences obtained in this study are indicated in black diamonds. [file Image_2.JPEG]
