# Supplementary material for: Molecular Detection of Zoonotic and Veterinary Pathogenic Bacteria in Pet Dogs and Their Parasitizing Ticks in Junggar Basin, North-Western China
Source: Front Vet Sci. 2022 Jul 8;9:895140. doi: 10.3389/fvets.2022.895140 (PMC9311330; doi:10.3389/fvets.2022.895140)
Supplement: Supplementary Table 1 — GenBank accession numbers of representative nucleotide sequences. [file Table_2.doc]

**Supplementary Table 1.** GenBank accession numbers of representative nucleotide sequences.

| **Location** | **Sample type** | **Gene** | **Accession number** | **Reference sequence** | **Similarity % (bp)** |
| --- | --- | --- | --- | --- | --- |
| **A** | | | | | |
| ***Rhipicephalus turanicus*** | | | | | |
| Shihezi | tick | 12S rRNA | MW067832 | *Rh. turanicus* Xinjiang-BC (MF002569) | 100 (400/400) |
| tick | COI | MW065551 | *Rh. turanicus* GY33 (MN853166) | 100 (707/707) |
| Shawan | tick | 12S rRNA | MW995983 | *Rh. turanicus* Xinjiang-BC (MF002569) | 100 (400/400) |
| tick | COI | MZ026893 | *Rh. turanicus* GY33 (MN853166) | 99.7 (659/661) |
| **B** | | | | | |
| ***Anaplasma* spp.** | | | | | |
| Shihezi | tick | 16S rRNA | MW757176 | *[Anaplasma](https://blast.ncbi.nlm.nih.gov/Blast.cgi#alnHdr_695166563)* sp. BL102-7 (KJ410249) | 100 (388/388) |
| blood | 16S rRNA | MW757177 | *[Anaplasma](https://blast.ncbi.nlm.nih.gov/Blast.cgi#alnHdr_695166563)* sp. BL102-7 (KJ410249) | 100 (388/388) |
| Shawan | tick | 16S rRNA | MZ048965 | *[Anaplasma](https://blast.ncbi.nlm.nih.gov/Blast.cgi#alnHdr_695166563)* sp. BL102-7 (KJ410249) | 99.23 (387/390) |
| blood | 16S rRNA | MZ048966 | *[Anaplasma](https://blast.ncbi.nlm.nih.gov/Blast.cgi#alnHdr_695166563)* sp. BL102-7 (KJ410249) | 99.74 (389/390) |
| ***Anaplasma phagocytophilum*** | | | | | |
| Shihezi | tick | 16S rRNA | MZ477261 | *A. phagocytophilum* ATS1 (KJ782386) | 99.84 (641/642) |
| blood | 16S rRNA | MZ477262 | *A. phagocytophilum* ATS1 (KJ782386) | 99.84 (641/642) |
| Shawan | tick | 16S rRNA | MZ477259 | *A. phagocytophilum* ATS1 (KJ782386) | 99.84 (641/642) |
| blood | 16S rRNA | MZ477260 | *A. phagocytophilum* ATS1 (KJ782386) | 99.84 (641/642) |
| ***Anaplasma ovis*** | | | | | |
| Shihezi | tick | msp4 | MW802667 | *A. ovis* 68 (MN198191) | 100 (776/776) |
| **C** | | | | | |
| ***Ehrlichia* spp.** | | | | | |
| Shihezi | tick | 16S rRNA | MW757178 | *Ehrlichia* sp. QYP9 ( [KY630175](https://www.ncbi.nlm.nih.gov/nucleotide/KY630175.1?report=genbank&log$=nuclalign&blast_rank=9&RID=E8G9U4T3016)) | 99.64 (280/281) |
| blood | 16S rRNA | MW757179 | *Ehrlichia* sp. QYP9 ( [KY630175](https://www.ncbi.nlm.nih.gov/nucleotide/KY630175.1?report=genbank&log$=nuclalign&blast_rank=9&RID=E8G9U4T3016)) | 99.64 (280/281) |
| Shawan | tick | 16S rRNA | MW990410 | *Ehrlichia* sp. XJ-Eh1 ([MF098393](https://www.ncbi.nlm.nih.gov/nucleotide/MF098393.1?report=genbank&log$=nuclalign&blast_rank=2&RID=E8GR2X2V013)) | 99.65 (281/282) |
| blood | 16S rRNA | MW990411 | *Ehrlichia* sp. XJ-Eh1 ([MF098393](https://www.ncbi.nlm.nih.gov/nucleotide/MF098393.1?report=genbank&log$=nuclalign&blast_rank=2&RID=E8GR2X2V013)) | 99.65 (281/282) |
| ***Ehrlichia chaffeensis*** | | | | | |
| Shihezi | tick | 16S rRNA | MZ540214 | *[E](https://blast.ncbi.nlm.nih.gov/Blast.cgi#alnHdr_1729880484). chaffeensis* (MN368552) | 99.23 (387/390) |
| blood | 16S rRNA | MZ540215 | *[E](https://blast.ncbi.nlm.nih.gov/Blast.cgi#alnHdr_1729880484). chaffeensis* (MN368552) | 99.23 (387/390) |
| Shawan | tick | 16S rRNA | MZ540216 | *[E](https://blast.ncbi.nlm.nih.gov/Blast.cgi#alnHdr_1729880484). chaffeensis* (MN368552) | 100 (390/390) |
| blood | 16S rRNA | MZ540217 | *[E](https://blast.ncbi.nlm.nih.gov/Blast.cgi#alnHdr_1729880484). chaffeensis* (MN368552) | 100 (390/390) |
| **D** | | | | | |
| ***Rickettsia massiliae*** | | | | | |
| Shihezi | tick | gltA | MZ020768 | *R. massiliae* Xinjiang-BC (MF002497) | 99.83 (1176/1178) |
| ompB | MW802695 | *R. massiliae* Xinjiang-BC (MF002502) | 100 (812/812) |
| Shawan | tick | gltA | MW802693 | *R.* *massiliae* Xinjiang-BC (MF002497) | 100 (1178/1178) |
| ompB | MW802696 | *R. massiliae* Xinjiang-BC (MF002502) | 100 (812/812) |
| blood | gltA | MZ020770 | *R. massiliae* Xinjiang-BC (MF002497) | 100 (1178/1178) |
| ompB | MZ020771 | *R. massiliae* Xinjiang-BC (MF002502) | 99.88 (811/812) |
| ***Rickettsia sibirica*** | | | | | |
| Shihezi | tick | gltA | MW802694 | *R. sibirica* Xinjiang-HBH (MF002540) | 100 (1178/1178) |
| ompB | MW802697 | *R. sibirica* Xinjiang-JMN (MF002551) | 99.38 (807/812) |
| Shawan | tick | gltA | MZ020772 | *R. sibirica* Xinjiang-HBH (MF002540) | 99.83 (1176/1178) |
| ompB | MZ020773 | *R. sibirica* Xinjiang-JMN (MF002551) | 100 (812/812) |
| **Candidatus Rickettsia barbariae** | | | | | |
| Shihezi | tick | gltA | MW802692 | Candidatus R. barbariae Xinjiang-YC (MF002503) | 100 (1101/1101) |
| ompB | MZ020774 | Candidatus R. barbariae Xinjiang-YC (MF002508) | 100 (827/827) |
| Shawan | tick | gltA | MZ020775 | Candidatus R. barbariae Xinjiang-YC (MF002503) | 100 (1101/1101) |
| ompB | MZ020776 | Candidatus R. barbariae Xinjiang-YC (MF002508) | 99.76 (825/827) |
| blood | gltA | MZ020777 | Candidatus R. barbariae Xinjiang-YC (MF002503) | 100 (1101/1101) |
| ompB | MZ020778 | Candidatus R. barbariae Xinjiang-YC (MF002508) | 99.88 (826/827) |
| **E** | | | | | |
| ***Brucella* spp.** | | | | | |
| Shihezi | tick | omp22 | MZ031925 | *Brucella* sp. YC31 (MK201679) | 100 (253/253) |
| blood | omp22 | MW802665 | *Brucella* sp. YC31 (MK201679) | 100 (253/253) |
| Shawan | tick | omp22 | MZ031926 | *Brucella* sp. YC31 (MK201679) | 100 (253/253) |
| blood | omp22 | MZ031927 | *Brucella* sp. YC31 (MK201679) | 99.60 (252/253) |
